# Grass cell wall feruloylation: distribution of bound ferulate and candidate gene expression in *Brachypodium distachyon*

**DOI:** 10.3389/fpls.2013.00050

**Published:** 2013-03-15

**Authors:** Hugo B. C. Molinari, Till K. Pellny, Jackie Freeman, Peter R. Shewry, Rowan A. C. Mitchell

**Affiliations:** Plant Biology and Crop Science, Rothamsted ResearchHarpenden, Hertfordshire, UK

**Keywords:** glucuronoarabinoxylan, hydroxycinnamic acid, BAHD gene family, PF02458 domain, bound phenolic

## Abstract

The cell walls of grasses such as wheat, maize, rice, and sugar cane, contain large amounts of ferulate that is ester-linked to the cell wall polysaccharide glucuronoarabinoxylan (GAX). This ferulate is considered to limit the digestibility of polysaccharide in grass biomass as it forms covalent linkages between polysaccharide and lignin components. Candidate genes within a grass-specific clade of the BAHD acyl-coA transferase superfamily have been identified as being responsible for the ester linkage of ferulate to GAX. Manipulation of these BAHD genes may therefore be a biotechnological target for increasing efficiency of conversion of grass biomass into biofuel. Here, we describe the expression of these candidate genes and amounts of bound ferulate from various tissues and developmental stages of the model grass *Brachypodium distachyon*. BAHD candidate transcripts and significant amounts of bound ferulate were present in every tissue and developmental stage. We hypothesize that BAHD candidate genes similar to the recently described *Oryza sativa* *p*-coumarate monolignol transferase (OsPMT) gene (PMT sub-clade) are principally responsible for the bound *para*-coumaric acid (*p*CA), and that other BAHD candidates (non-PMT sub-clade) are responsible for bound ferulic acid (FA). There were some similarities with between the ratio of expression non-PMT/PMT genes and the ratio of bound FA/*p*CA between tissue types, compatible with this hypothesis. However, much further work to modify BAHD genes in grasses and to characterize the heterologously expressed proteins is required to demonstrate their function.

## INTRODUCTION

Many of the most abundant potential lignocellulosic feedstocks are from grasses, whether by-products from food crops such as wheat straw, rice straw, maize stover, and sugar cane residues or from specialized bioenergy crops such as *Miscanthus* and switch grass. Such feedstocks differ from those from dicotyledonous crops in the occurrence of *trans*-ferulic acid (FA), a hydroxycinnamic acid which is ester-linked to the hemicellulosic component, glucuronoarabinoxylan (GAX; Carpita, 1996; Scheller and Ulvskov, 2010). This linkage occurs only in commelinid monocots and is particularly abundant in grasses (Harris and Trethewey, 2010). It is considered to affect digestibility (Grabber, 2005; Dhugga, 2007), as FA can oxidatively cross-link to form ether bonds or C–C bonds, linking chains of GAX (Ishii, 1991), or of GAX to lignin (Ralph et al., 1995). Thus it covalently links the cell wall polysaccharide which can be used as a substrate for biofuel to the inhibitory lignin component. Whilst the barrier to degradation presented by FA is mainly structural, FA also has antimicrobial activity, inhibiting sugar fermentation by yeast (Baranowski et al., 1980; Akin, 2008). Grasses with decreased feruloylation of GAX in their cell walls may therefore be more readily converted into biofuel. In particular, they will have a lower energy requirement for separation of lignin from polysaccharide, an essential step in most pipelines for biomass processing.

The genes and enzymes responsible for feruloylation of GAX are still unknown. However, using a bioinformatics analysis of publicly available expressed sequence tags (ESTs), we identified an orthologous group within the BAHD acyl-coA superfamily as potential candidates based on their differential expression in cereals compared with dicots (Mitchell et al., 2007). The BAHD family (also referred to as the PF02458 family, as all family members contain the PFAM domain PF02458 which is specific to the family) is named after the first four members to be biochemically characterized (benzylalcohol acetyltransferase, BEAT; anthocyanin hydroxycinnamoyl transferase, AHCT; anthranilate hydroxycinnamoyl/benzoyl transferase, HCBT; deactylvindoline acetyltransferase, DAT; D’Auria, 2006). The predicted role in GAX feruloylation is compatible with the several hydroxycinnamoyl transferase activities creating ester bonds known in the family, with feruloyl-CoA acting as the donor. We also identified GT61 genes as likely to be involved in GAX biosynthesis (Mitchell et al., 2007), and have recently demonstrated (in collaboration with the Dupree lab, University of Cambridge, UK) that these genes mediate the addition of 3-linked arabinofuranose (Ara*f*) to xylan (Anders et al., 2012). GT61 genes tend to be co-expressed with the BAHD candidates in cereals (Mitchell et al., 2007; Shewry et al., 2011), consistent with a role in feruloylation of the 3-linked Ara*f* residues added by GT61 proteins. Our predicted role of BAHD genes in feruloylation was tested by another group using RNAi suppression in rice, showing a modest (-19%) but significant decrease in cell wall bound ferulate in the stems of the transgenic plants (Piston et al., 2010). However, this effect could be an indirect consequence of gene suppression, so the role of these BAHD genes still requires elucidation.

One question is the intracellular localization of the proteins encoded by the BAHD genes: this is predicted to be cytosolic based on the protein sequences and this is the localization for all known members of the family (D’Auria, 2006), whereas GAX is synthesized in the Golgi (Buanafina, 2009). An activity from rice seedlings capable of feruloylation of Ara*f* on a synthetic molecule was found in the soluble fraction rather than the membrane fraction, consistent with a cytosolic enzyme (Yoshida-Shimokawa et al., 2001). A possible explanation is that BAHD proteins are responsible for feruloylation of a cytosolic precursor, such as UDP-Ara*f*, which is the substrate for xylan arabinosylation. Whereas the great majority of UDP-arabinopyranose is synthesized in the Golgi lumen, this is converted by a mutase to the UDP-Ara*f* form in the cytosol (Konishi et al., 2007; Rautengarten et al., 2011) so this is a possible substrate for a cytosolic feruloylation reaction *in planta*. The product from this would then be transported back into the Golgi, where feruloylated Ara*f* would be transferred onto GAX, possibly by a GT61 enzyme. The feruloylation of arabinoxylan has been shown to occur within the protoplast (Myton and Fry, 1994), although there is also evidence of an alternative pathway which allows feruloylation in the wall when inhibitors are used to suppress the secretory pathway (Mastrangelo et al., 2009).

A key recent breakthrough was the demonstration that at least one of these BAHD genes in rice has a different role from that predicted; in that the encoded protein catalyzed the addition not of FA to a sugar, but of the closely related *para*-coumaric acid (*p*CA) to monolignols. This gene therefore encodes a *p*CA monolignol transferase and has been named OsPMT (*Oryza sativa* *p*-coumarate monolignol transferase; Withers et al., 2012). Interestingly, whereas other BAHD proteins have been shown to use both FA-CoA and *p*CA-CoA as donor molecules (Luo et al., 2007), OsPMT was found to have almost no activity with FA-CoA (Withers et al., 2012). Whereas it is theoretically possible that OsPMT could also add FA or *p*CA to Ara*f*, activities with such different acceptors have not previously been found for BAHD proteins. OsPMT activity is presumably responsible for forming ester linkages between *p*CA and lignin, a linkage which is also much more common in grasses than in other groups of plants. It is therefore possible that the candidate group which includes OsPMT is responsible only for this activity, rather than also for GAX feruloylation. However, to date, only one of the genes has been shown to have this activity and others are expressed (albeit at low levels) in wheat starchy endosperm which does not have lignin but does have feruloylated AX (Pellny et al., 2012), suggesting that other genes within the group are responsible for xylan feruloylation. During preparation of this article, strong support for this theory was published where another BAHD gene within this group was overexpressed in rice, resulting in increased bound *p*CA with good evidence that this was ester-linked to GAX, not lignin (Bartley et al., 2013). Whereas this gene seems specific for *p*CA, it seems very likely that some of the other similar genes in the clade are responsible for FA ester-linked to GAX.

*Brachypodium distachyon* is a model grass species which has a small and fully sequenced genome, a short life cycle, and small size, making it ideal for the study of grass cell walls (Vogel et al., 2010). We have therefore determined the contents of bound FA and *p*CA (predominantly linked to GAX and lignin, respectively) in various tissues and at different stages throughout the life cycle, and related these to the expression of BAHD genes identified as candidates for the addition of these phenolic acids to cell wall polymers (GAX and lignin).

## RESULTS AND DISCUSSION

**FIGURE 1:**
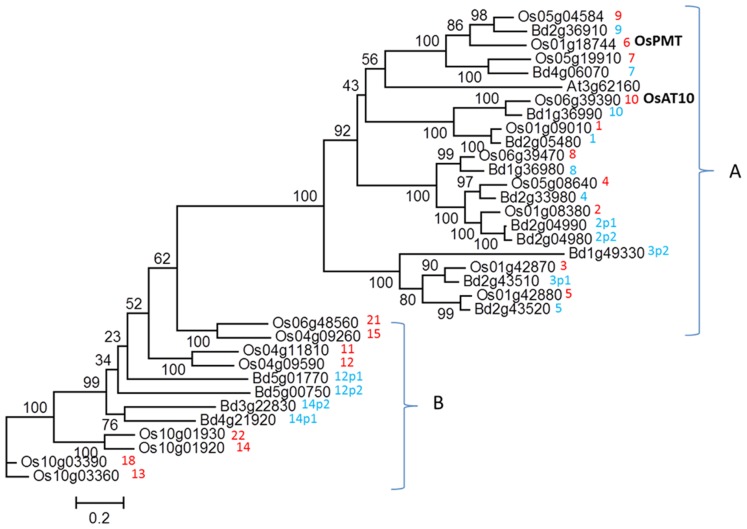
**Phylogenetic tree of clades within BAHD gene superfamily for rice and *Brachypodium* genes, which were identified as containing potential candidates for xylan feruloylation (Mitchell et al., 2007)**. Numbers are arbitrary candidate numbers based on rice genes; where two paralogous *Brachypodium* genes match one rice gene these are given the same number but with “p1” or “p2” appended to the name. Genes within clade A are generally more highly expressed than those in clade B. The recently identified monolignol *p*CA transferase *Os*PMT (Withers et al., 2012) and OsAt10 (involved in *p*CA addition to xylan; Bartley et al., 2013) genes are indicated.

**Figure 1** shows a phylogenetic tree of the clades of the BAHD gene superfamily which have been proposed to contain candidate genes for the feruloylation of GAX in grass cell walls (Mitchell et al., 2007), divided into clades A and B. Clade B genes are not highly expressed or co-expressed with xylan pathway genes, and do not have clear orthologs between rice and *Brachypodium*. The more highly expressed clade A genes were therefore identified as the stronger candidates (Mitchell et al., 2007) for AX feruloylation. Clade A also includes the gene subsequently shown to encode a monolignol *p*CA transferase in rice, OsPMT (Withers et al., 2012). Two wheat genes, orthologous to candidates 1 and 3, are expressed in wheat starchy endosperm, which does not contain lignin in the cell walls (Pellny et al., 2012). An RNAi construct which simultaneously suppressed the expression of rice candidates 1, 4, 8, and 10 (in our nomenclature) resulted in a decrease in cell wall FA of 19% in transgenic rice (Piston et al., 2010). A possible interpretation is therefore that the monolignol *p*CA transferase activity is restricted to the sub-clade containing OsPMT which includes candidates 7 and 9 and the only *Arabidopsis* gene, and the other candidates in clade A may be involved in GAX feruloylation. This is supported by the recent demonstration that overexpressing OsAt10 (coincidentally also candidate 10 in our nomenclature) resulted in increased *p*CA almost certainly ester-linked to GAX (Bartley et al., 2013). This ester linkage is less common than FA in most tissues and is absent in wheat starchy endosperm. From all the evidence, we hypothesize that the activity responsible for GAX feruloylation will exist in some or all of the candidates 1–5 and 8.

**FIGURE 2:**
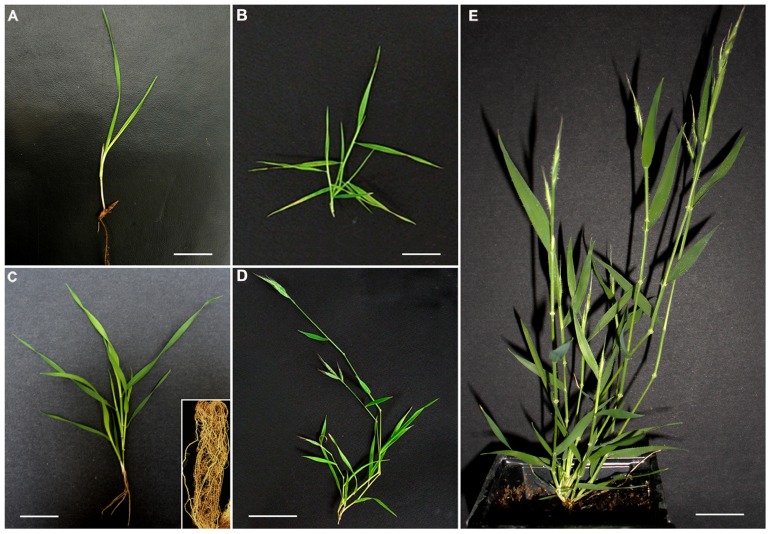
***Brachypodium* developmental stages**. **(A)** Early vegetative phase (EVP), bar = 1cm; **(B)** late vegetative phase (LVP), bar = 2cm; **(C)** transition phase (TP), bar = 2cm; **(D)** reproductive phase (RP), bar = 3cm; **(E)** advanced phase (AP), bar = 2cm.

The expression of candidate genes and amounts of bound FA and *p*CA were determined in tissues of developing *B. distachyon* plants as shown in **Figure 2**.

**FIGURE 3:**
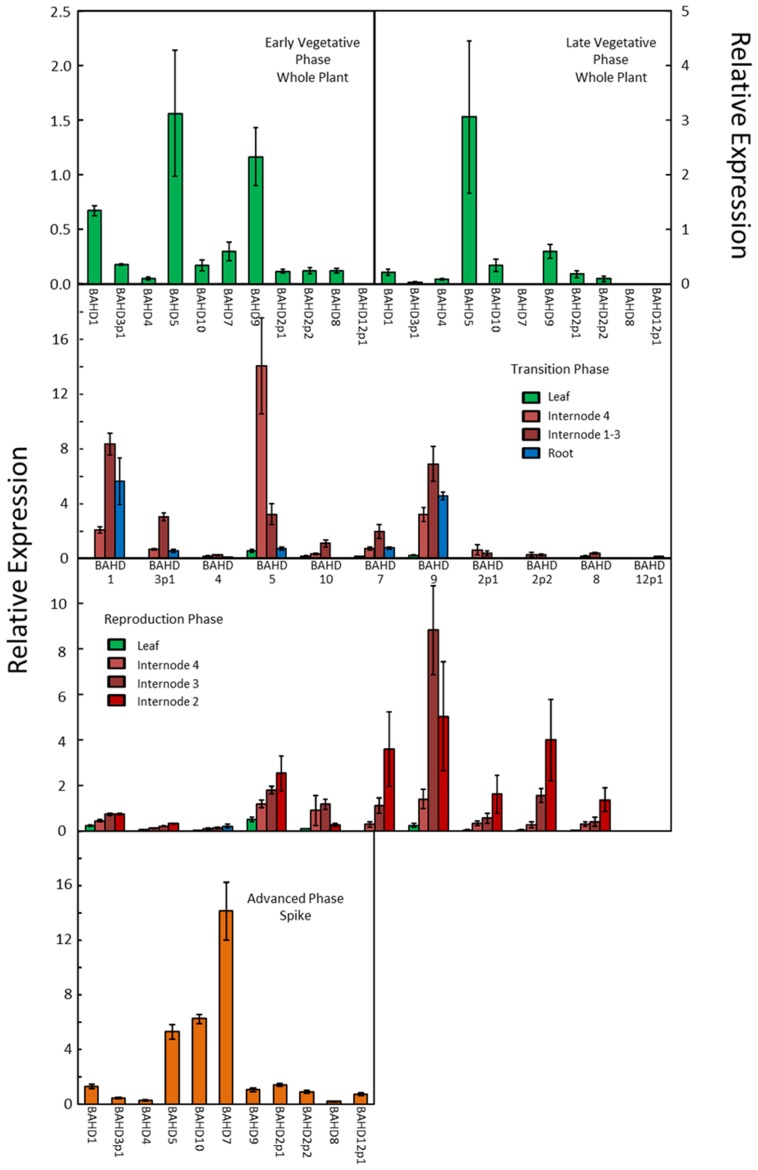
**Transcript abundance of candidate BAHD genes in developing *Brachypodium* tissues estimated by qRT-PCR**. Error bars are ±SE from three biological replicates. One-way analysis of variance showed that the effect of tissue/stage on transcript abundance was significant at *F* probability<0.001 for every gene except BAHD2p1, where it was significant at *F* probability = 0.002.

The transcript abundances of the BAHD genes (denoted by the candidate numbers shown in **Figure 1**), were determined by quantitative real-time RT-PCR (qRT-PCR) and the values are shown for the different genes, tissues, and developmental stages in **Figure 3**. The primer efficiencies were comparable for the different genes (**Table 2**), so differences in the abundances determined for the genes should reflect real differences in transcript abundances. On this basis, it can be concluded that the BAHD gene candidates which are most highly expressed in above-ground tissue samples from the vegetative phase are, in descending order of abundance, 5, 9, 1, whereas in roots they are 1, 9. In later stages of reproductive development, they are 9, 7, 5, and 2p2, and in the spike at the advanced stage 7, 10, 5 (**Figure 3**). Therefore, BAHD genes from clade A are highly expressed in all tissues that were analyzed. Based on the phylogenetic relationship with *Os*PMT (**Figure 1**) and the assumption that *Bd*BAHD 9 and 7 are responsible for the addition of coumaryl esters on lignin (i.e., PMT activity), and the others responsible for xylan feruloylation (with the exception of 10 which is more likely responsible for coumaryl esters on xylan), it can be noted that at least one gene with each function is highly expressed in every tissue that was analyzed, with the genes with PMT activity being relatively more highly expressed than other BAHD candidate genes in the reproductive growth phase.

**FIGURE 4:**
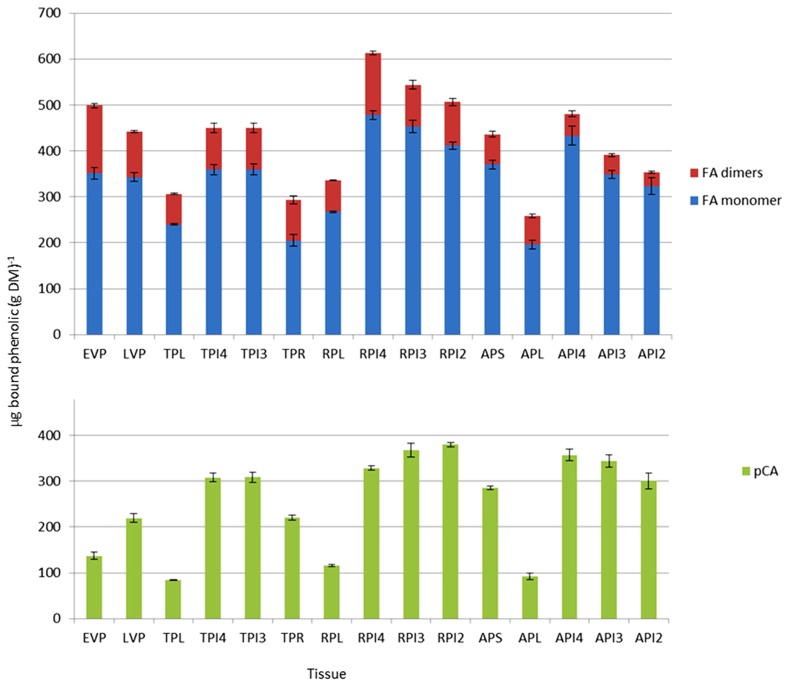
**Bound hydroxycinnamic acid content from developing *Brachypodium* tissues estimated by HPLC**. Samples were early vegetative plants (EVP), late vegetative plants (LVP), transition phase leaves (TPL), transition phase internodes (TPI4, TPI3), transition phase roots (TPR), reproductive phase leaves (RPL), reproductive phase internodes (RPI4, RPI3, RPI2), advanced phase spike (APS), advanced phase leaves (APL), and advanced phase internodes (API4, API3, API2). FA dimers are the sum of the four major dimers described in Section “Materials and Methods.” Error bars are ±SE from three biological replicates. One-way analysis of variance showed that the effect of tissue/stage on both total FA and pCA content was significant at *F* probability<0.001.

Determination of amounts of bound phenolic acids in plant samples is a measure of the amounts that are covalently linked to the cell wall fraction. Protocols using moderate alkaline treatment release only the ester-linked phenolics from cell walls, which are then separated and quantified by high-performance liquid chromatography (HPLC). By far the most abundant bound phenolic acids are FA and *p*CA. FA is considered to be exclusively linked to GAX by ester bonds in grass cell walls, while the *p*CA is predominantly ester-linked to lignin, with a much lower amount ester-linked to GAX. FA, unlike *p*CA, can oxidatively couple *in planta* to form dimers or higher-order oligomers. The amounts of bound FA monomer, major FA dimers and bound *p*CA are shown for different tissues in **Figure 4**. The amount of *p*CA reflects principally the amount of this phenolic acid which is ester-linked to lignin, and this fraction has been found to be highly correlated with the degree of lignification in grasses (Grabber et al., 2004). The values shown are therefore consistent with increased lignification during the reproductive phase, relative to GAX feruloylation, which occurs in both primary and secondary grass cell walls. The absolute amounts of FA and *p*CA in young shoots (early vegetative phase, EVP) determined here are also comparable with those determined for seedlings at 8 days after germination (DAG) where FA and *p*CA were found to be ~400 and ~200μgg^-1^, respectively (Christensen et al., 2010) and with a recent analysis of more tissues and stages (Rancour et al., 2012). A consistent feature of the data presented here (**Figure 4**) and by Rancour et al. (2012) is that the *p*CA:FA ratio is higher in stems compared to leaves, which is consistent with the comparatively greater lignification in stems.

**FIGURE 5:**
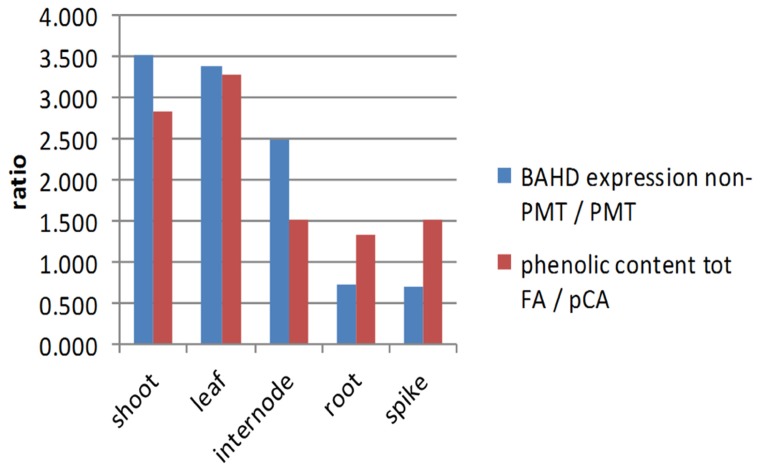
**Ratios of expression of non-PMT BAHD to PMT BAHD and of bound FA to *p*CA**. Expression ratio is the sum of values for genes 1–5 and 8 (non-PMT) relative to the sum of values for genes 7 and 9 (PMT). FA to pCA ratio is the sum of FA monomer and dimers relative to *p*CA. Ratios shown are averaged over EVP, LVP for shoots, TPL, RPL for leaves, TPI4, RPI4, RPI3, RPI2 for internodes; root is TPR and spike is APS.

Expression of BAHD candidates from both the PMT and non-PMT sub-clades was found in every tissue examined (**Figure 3**), as was substantial quantities of bound FA and *p*CA (**Figure 4**). Simple correlations are not expected between the abundances of transcripts encoding enzymes in a biosynthetic pathway and the amount of end-product from that pathway. Transcript abundance measures the potential for enzyme synthesis only at a single time point whereas end-product accumulation occurs throughout a developmental process and may be limited by substrate availability as well as enzyme activity, which in turn depends on enzyme activation as well as enzyme amount. However, tissues which tend to have more bound FA compared to total bound *p*CA, might also be expected to have greater expression of BAHD candidates in the non-PMT sub-clade relative to those in the PMT sub-clade. We found this to be broadly the case, with shoots during vegetative phase and leaves in later phases, which have high bound total FA top *p*CA content (due to less lignification), also having greater ratios of non-PMT to PMT expression than internodes, roots, and spike (**Figure 5**).

We have previously identified co-expression between BAHD candidates and xylan synthesis genes, compatible with a role in GAX feruloylation (Mitchell et al., 2007; Shewry et al., 2011), but given the new findings on these candidate genes (Withers et al., 2012; Bartley et al., 2013), we decided to re-examine this. Public resources are not yet extensive for *Brachypodium* gene expression, but using the RiceFREND tool which employs a wide range of rice transcriptome experiments (Sato et al., 2013), we examined co-expression for the rice BAHD genes with rice genes putatively involved in xylan synthesis (**Table 1**). Nearly all the clade A genes had xylan synthetic genes in the top 1% of genes ranked by expression correlation, but none of the clade B did. BAHD1, 5, and 8 showed particularly close co-expression with genes involved in UDP-Xyl and UDP-Ara synthesis and GT61 genes, whereas OsPMT and similar genes (BAHD7 and BAHD9) had fewer closely co-expressed genes. If these genes in the PMT sub-clade are responsible for addition of *p*CA to lignin then some co-expression may be expected with any genes involved in secondary cell wall xylan synthesis.

**Table 1:** Co-expression of BAHD candidate genes in rice with genes putatively involved in xylan synthesis.

	BAHD candidate clade		A										B			
		Candidate no.	1	2	3	4	5	6	7	8	9	10	12	14	15	18
Putative role	Family	MSU locus	Os01g09010	Os01g08380	Os01g42870	Os05g08640	Os01g42880	Os01g18744 PMT	Os05g19910	Os06g39470	Os05g04584	Os06g39390 OsAt10	Os04g09590	Os10g01920	Os04g09260	Os10g03390
UDP-Xyl and UDP-Ara synthesis	UDP-Glc dehydrogenase	Os03g55070	237		15		6									
	UDP-Glc dehydrogenase	Os12g25690			244		33									
	UDP-GlcA decarboxylase	Os01g21320									123					
	UDP-GlcA decarboxylase	Os01g62020		98								119				
	UDP-GlcA decarboxylase	Os03g16980			90		7					73				
	UDP-GlcA decarboxylase	Os05g29990	21	76	56											
	UDP-Xyl epimerase	Os07g04690	88							76						
	UDP-Xyl epimerase	Os04g52730	24	166												
UDP-Araf synthesis	UDP-Ara mutase	Os03g40270			67		66									
	UDP-Ara mutase	Os07g41360					62									
Xylan backbone synthesis	GT family 43	Os01g48440								238						
	GT family 43 IRX9-like	Os03g17850										148				
	GT family 43	Os04g55670		246			71			194		30				
	GT family 43	Os05g03174			62		57									
	GT family 43	Os05g48600		129						38						
	GT family 43 IRX14-like	Os06g47340	197	200						10		139				
	GT family 47 IRX10-like	Os01g70200		203			250	198		142		70				
Araf addition to xylan	GT61 clade A	Os01g02900				223	160	187			240					
	GT61 clade A	Os01g02930		248												
	GT61 clade A	Os02g04250	4	175			58			63						
	GT61 clade A	Os02g22380	14	36			45			11		136				
	GT61 clade A	Os06g27560						58			57					
	GT61 clade A	Os06g49300										175				

*Values are the mutual rank from the RiceFREND co-expression tool (http://ricefrend.dna.affrc.go.jp/;Sato et al., 2013) using the BAHD candidate under single guide gene search, out of the 27,201 genes in the database; only ranks <250 are shown, i.e., in the top 1% of the most highly co-expressed genes. Some clade B genes shown in **Figure 1** are omitted as they were not in the RiceFREND database.*

## CONCLUSION

The genes identified as candidates for being involved in feruloylation of GAX (Mitchell et al., 2007) are expressed in every tissue and at every developmental stage in the model grass *Brachypodium* as expected for a process which is required for every primary and secondary cell wall in the plant. The relative amounts of *p*CA and FA in these different tissues seem compatible with the expression of BAHD candidate genes, where these are divided into those putatively responsible for *p*CA linked to lignin (PMT) and FA linked to xylan (non-PMT). The evidence presented here does not demonstrate function; for this more studies with targeted modification of the genes in grass species (as in Bartley et al., 2013) and *in vitro* characterization of pure proteins (as in Withers et al., 2012) are required. If their role in feruloylation is confirmed by such experiments, then as feruloylation is believed to be key to the exploitation of grass biomass for biofuel and animal feed, these genes would represent important biotechnological targets.

## MATERIALS AND METHODS

### PLANT GROWTH

*Brachypodium* plants Bd21 were grown in standard glass house conditions at 25°C. The whole aerial plant was harvested for the EVP (7 DAG) and late vegetative phase (LVP; 12 DAG). Plants were vernalized at 4°C for 2 weeks during the EVP; references to “DAG” do not include this period. In the transition (20 DAG) and reproductive (30 DAG) phases, the plants were separated into leaf and internode tissues, whereby internode 1 is the oldest and 4 the upmost and youngest. In the transition phase, roots were also harvested. Spikelets, i.e., the developing seeds with the surrounding maternal tissues, were harvested in the advanced phase (50 DAG). Material was immediately frozen in liquid nitrogen and stored at-80°C.

### PHYLOGENETIC ANALYSIS

Protein sequences for the whole BAHD superfamily were identified as all rice, *Arabidopsis*, and *Brachypodium* sequences from Phytozome^1^ containing the PFAM domain PF02458. An initial tree was generated and all sequences contained within the candidate group identified in Mitchell et al. (2007) were aligned with the MUSCLE algorithm (Edgar, 2004). Gapped columns were removed, followed by phylogeny analysis of aligned sequences in the PhyML package (Guindon et al., 2005) using the Whelan and Goldman (2001) model. An initial run optimized the gamma and invariant proportion parameters; these were then held constant for 100 runs for bootstrap non-parametric analysis and the maximum likelihood tree is presented.

### DETERMINATION OF TRANSCRIPT ABUNDANCE

RNA was extracted using a cetyltrimethylammonium bromide (CTAB) method following Chang et al. (1993). Quantitative PCR was performed as in Pellny et al. (2008) on an Applied Biosystems 7500 real-time PCR system^2^ using SYBR green JumpStart Kit (Sigma-Aldrich^3^) following the manufacturer’s instructions. The expression of the genes of interest was normalized with two endogenous controls ubiquitin-conjugating enzyme 18 (UBC18) and succinate dehydrogenase (SDH). Putative house-keeping genes glyceraldehyde-3-phosphate dehydrogenase (GAPDH), *S*-adenosylmethionine decarboxylase (SAMDC), and peptide deformylase 2 (PDF2) were also tested but found less stable over the different tissue and time points. Expression calculations were performed as suggested by Rieu and Powers (2009). Individual amplification efficiencies were established with LinRegPCR using a window-of-linearity and the relative quantities (RQs) for the target genes were calculated. This was normalized with the geometrical mean of the RQs of the control genes. Primers used and average efficiency of pairs are listed in **Table 2**.

**Table 2:** PCR primers.

Oligo name	Orientation	Sequence (5′–3′)	Target	Average primer efficiency
prHM01	Sense	GCCGCACAACACCATCATG	qRT-PCR Amplicon Bd_Bahd_1	1.74
HM02	Antisense	GGCTTTGATGAACTGCGCC	qRT-PCR Amplicon Bd_Bahd_1	
HM07	Sense	ACCTCATCCTCATGGCCCA	qRT-PCR Amplicon Bd_Bahd_3p1	1.745
HM08	Antisense	CGAAAACCAGGTGGCTGAAG	qRT-PCR Amplicon Bd_Bahd_3p1	
HM09	Sense	CCGGTAAGGTGGCCTCGT	qRT-PCR Amplicon Bd_Bahd_4	1.84
HM10	Antisense	CGAACTCTGAAGGCAGCCG	qRT-PCR Amplicon Bd_Bahd_4	
HM11	Sense	CGTTCACCGCTTTCAACTTTG	qRT-PCR Amplicon Bd_Bahd_5	1.72
HM12	Antisense	TCGCAACCTGGTCTTTGACAC	qRT-PCR Amplicon Bd_Bahd_5	
HM19	Sense	CCGGTGCTAGCCCTGGAATA	qRT-PCR Amplicon Bd_Bahd_10	1.77
HM20	Antisense	TGCACGCGTTGTACTCCGA	qRT-PCR Amplicon Bd_Bahd_10	
HM33	Sense	CGCAAGACAATGACCGCTATG	qRT-PCR Reference gene Bd_UBC18	1.78
HM34	Antisense	CCAATCCGACGCCTCCTTATA	qRT-PCR Reference gene Bd_UBC18	
HM35	Sense	TGTTTGTGTCGGATTGGACGA	qRT-PCR Amplicon Bd_Bahd_7	1.69
HM36	Antisense	ACCGCCATGTAGTCCGCATAA	qRT-PCR Amplicon Bd_Bahd_7	
HM43	Sense	TTCTCGTATCACCCCTTCATGG	qRT-PCR Amplicon Bd_Bahd_9	1.75
HM44	Antisense	GGTGGTCTTCCTCCACACACAT	qRT-PCR Amplicon Bd_Bahd_9	
HM55	Sense	TGGCTTCTACGGCAACTGCTA	qRT-PCR Amplicon Bd_Bahd_2p1	1.79
HM56	Antisense	GCTTCCCGTCCTTGATGATCT	qRT-PCR Amplicon Bd_Bahd_2p1	
HM61	Sense	AAGCGGCTCGAGTACCTG	qRT-PCR Amplicon Bd_Bahd_2p2	1.82
HM62	Antisense	GCCATTGTTGCTGGAGTTGT	qRT-PCR Amplicon Bd_Bahd_2p2	
HM65	Sense	CCACGTCTGCTTCGCCATG	qRT-PCR Amplicon Bd_Bahd_8	1.79
HM66	Antisense	CGCATGATGTAGTAGCAGTTGCC	qRT-PCR Amplicon Bd_Bahd_8	
HM69	Sense	ATCCCGCCATCCAACATCTAC	qRT-PCR Amplicon Bd_Bahd_12	1.79
HM70	Antisense	CGGATCTGGCCTTGATGTTGT	qRT-PCR Amplicon Bd_Bahd_12	
HM75	Sense	TTCAACAGCATGGATGGCC	qRT-PCR Reference gene Bd_SDH	1.76
HM76	Antisense	ATCTTCGGTTGCAGAGCTCCT	qRT-PCR Reference gene Bd_SDH	

### DETERMINATION OF BOUND PHENOLIC CONTENT

Cell wall bound phenolics were released by alkaline hydrolysis of alcohol insoluble residues (AIRs) from samples (20mg) of freeze-dried, ground tissue and extraction into ethyl acetate as previously described (Pellny et al., 2012). 3,5-Dichloro-4-hydroxybenzoic acid (20μL at 1.5mgmL^-1^) was added to AIR of all samples, prior to alkaline hydrolysis, as an internal standard. Samples were dissolved in 1mL 50% methanol:2% acetic acid (v:v) and bound phenolic acids from 40μL of extract separated by HPLC on a Shimadzu Prominence high-performance liquid chromatograph as described by Waldron et al. (1996) but using a binary gradient pump system. Quantitation of FA and *p*CA was by integration of peak areas at 280nm with reference to calibrations made using known amounts of pure compounds. Peaks of the major FA dimers were identified by comparison of retention times with pure standards kindly supplied by Professor John Ralph (Lu et al., 2012; 5-5, 8-*O*-4, 8-5-benzofuran) or by comparison of spectrum with that in Waldron et al. (1996) (8-5). Dimer quantitation was achieved relative to FA monomer using areas of these peaks and the response factors for dimers and FA monomer in Waldron et al. (1996). All samples were extracted and analyzed in triplicate.

## Conflict of Interest Statement

The authors declare that the research was conducted in the absence of any commercial or financial relationships that could be construed as a potential conflict of interest.

## Footnotes

1 www.phytozome.org

2 www.appliedbiosystems.com

3 www.sigmaaldrich.com
